# Supplementary figures and images for: Adipocyte-Specific Ablation of PU.1 Promotes Energy Expenditure and Ameliorates Metabolic Syndrome in Aging Mice
Source: Front Aging. 2022 Feb 2;2:803482. doi: 10.3389/fragi.2021.803482 (PMC9261351; doi:10.3389/fragi.2021.803482)

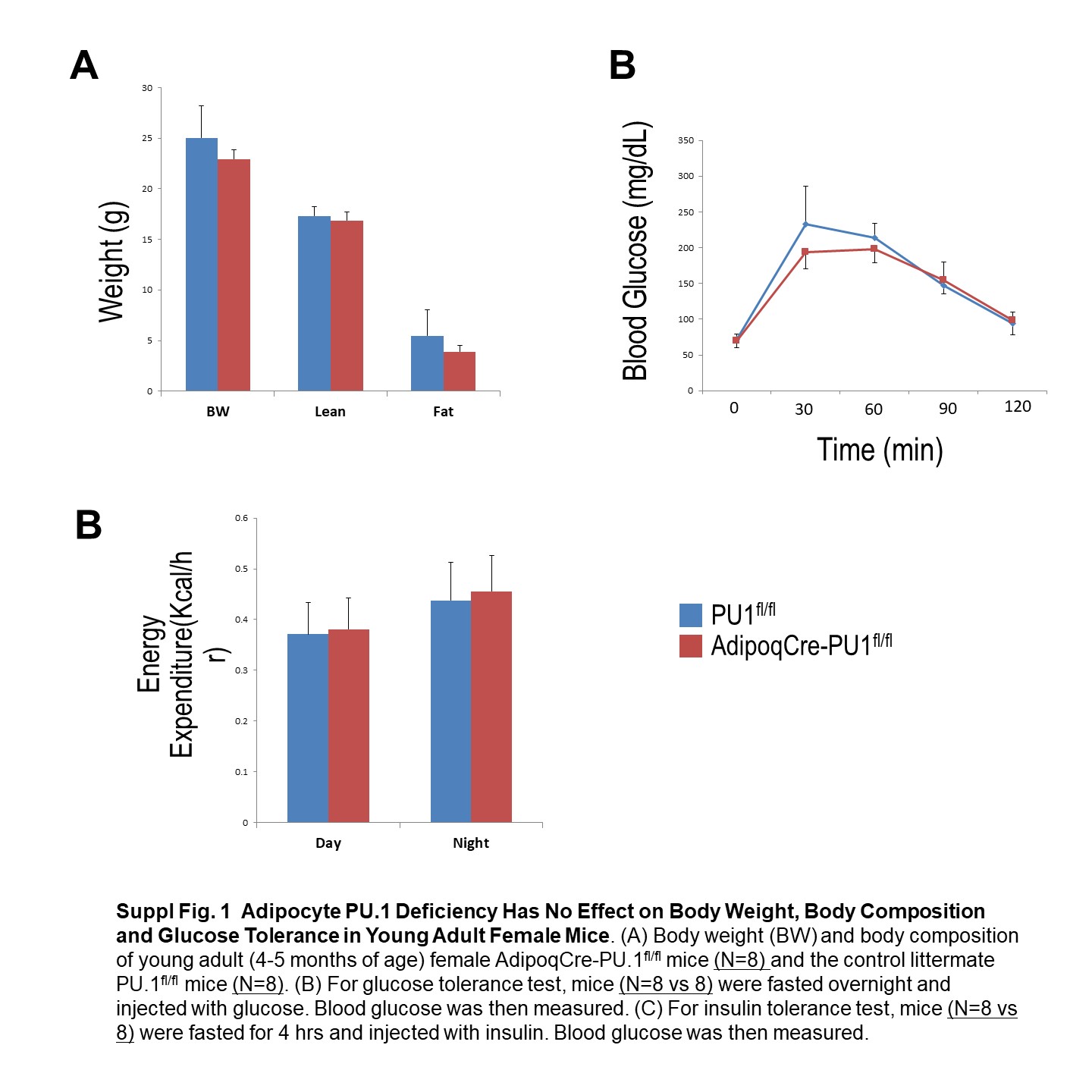

Supplement: Supplementary file 3 [file Image1.JPEG]

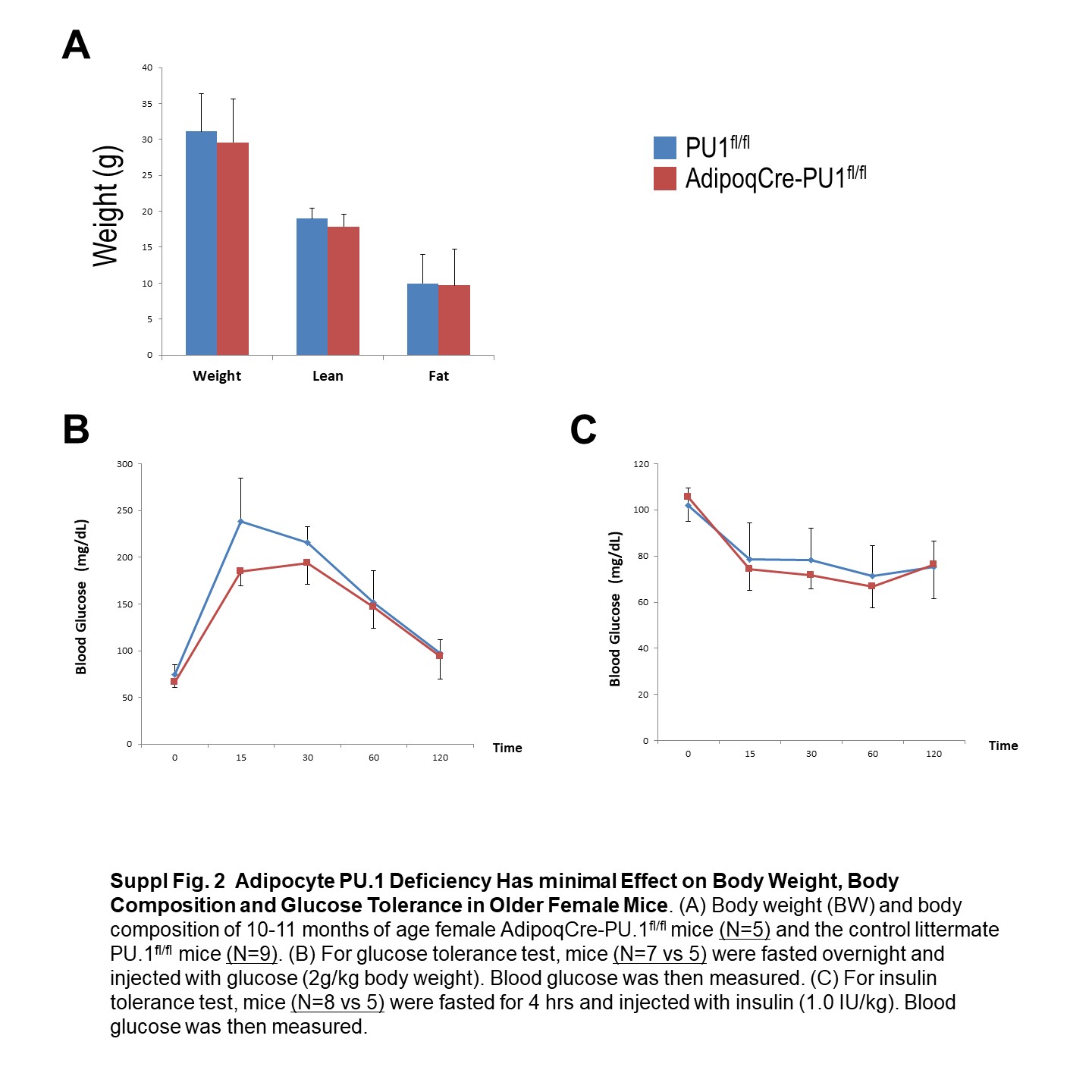

Supplement: Supplementary file 4 [file Image2.JPEG]
